# Deep brain stimulation for dystonia

**DOI:** 10.1055/s-0043-1767763

**Published:** 2023-04-14

**Authors:** Brendan Santyr, Renato P. Munhoz, Andres M. Lozano

**Affiliations:** 1 University of Toronto, Toronto Western Hospital, Division of Neurosurgery, Toronto, Ontario, Canada.; 2 University of Health Network, Morton and Gloria Shulman Movement Disorders Clinic, Edmond J. Safra Program in Parkinson's Disease, Toronto, Ontario, Canada.; 3 University of Toronto, Toronto Western Hospital, Division of Neurology, Toronto, Ontario, Canada.; 4 Krembil Research Institute, Toronto, Ontario, Canada.

Dystonia syndromes are diverse movement disorders characterized by disabling, painful, and sustained involuntary muscle contraction. 1 It's classified by body distribution (focal, segmental, multifocal, hemidystonia, and generalized) and etiology (heritable, secondary to nervous system pathology, or idiopathic). 1 The pathophysiology is poorly understood but lesion studies, 2 functional imaging, 3 and electrophysiological studies 4 provide evidence for the involvement of the basal ganglia, specifically the globus pallidus. Symptoms result from impaired sensory-motor inhibition resulting in increased basal ganglia excitability and decreased spatial and temporal somatosensory discrimination. 5 Treatment is aimed at reducing pain and functional impairment, with pharmacological approaches as the first line. Despite multiple options, many patients with generalized and some focal dystonias remain refractory to pharmacological treatments. 6

Various neurosurgical interventions have been trialled for the treatment of dystonia, including peripheral denervation, intrathecal baclofen infusion, and ablating the basal ganglia or thalamus. However, deep brain stimulation (DBS) primarily targeting the globus pallidus internus (GPi) and the subthalamic nucleus (STN) has evolved as a covetable option with the ability to provide personalized, reversible, and titratable neuromodulation. The available literature has recently been reviewed 7 and demonstrates the safe and efficacious use of DBS in dystonia for both GPi or STN, resulting in excellent and equivalent improvement in patients' movement and disability scores. Efficacy was also assessed relative to the body distribution with focal dystonia exhibiting better improvement in motor symptoms scores but less enhancement in quality of life compared with segmental dystonia. However, all dystonia distributions studied showed significant postoperative improvement. Also, it is well established that those with primary dystonias also respond better to DBS than those with secondary dystonias. Moreover, those with motor improvement from DBS will likely also demonstrate significant improvement in disability symptoms. As such, the current state of the literature supports the use of DBS, however, still fails to explain outcome variability and does not allow for a tailored patient-specific approach to management with DBS. Advances in neuroimaging may provide insights into this through patient-specific treatment selection, surgical targeting, and DBS programming.

In the current edition of the Arquivos de Neuro-Psiquiatria, Listik et al. 8 present work aimed at better understanding factors that predict individual patient responsiveness to DBS therapy. They hypothesize that connectivity of the stimulation site may be responsible for some of the DBS response. Motor impairment and disability scores (Burke-Fahn-Marsden Dystonia Rating Scale) were prospectively acquired in 5 patients with generalized dystonia of inherited/idiopathic etiology and undergoing bilateral STN-DBS for refractory motor symptoms. Using a combination of stimulation-outcome mapping and normative connectivity analysis, the authors show that stimulation location within the STN target does not explain the variability in clinical outcomes seen. However, the pattern of connectivity between the stimulated region and the left pre and postcentral gyrus and right cerebellar lobule III and vermis IX were significantly correlated with improved motor response to DBS. This supports other recent evidence that states the ideal DBS target relies not only on the anatomical position but also on its structural connectivity. 9 This work presents a step toward individualized target selection based on preoperative patient characteristics, including structural connectivity.

Similarly, intraoperative target selection has been further refined by studies employing stimulation-outcome mapping in large cohorts. Elias et al. 10 examined the motor outcome in 64 dystonia subjects (11 idiopathic/genetic, 53 acquired) who underwent bilateral GPi-DBS. They showed the greatest symptomatic improvement with stimulation in the ventroposterior GPi gray matter, located 3mm posterior, 1mm superior, and 1mm medial to the typical target location. They also localized areas of stimulation associated with poorer response to the superior parts of the external globus pallidus. These maps can predict clinical variance in outcomes following DBS therapy and provide preliminary blueprints for refined surgical targeting and postoperative DBS programming.

Insights into outcome variation in DBS therapy have also come from the functional magnetic resonance imaging literature. Loh et al. demonstrate that optimal DBS programming engages a functional network resulting in sensorimotor cortex deactivation in 15 cervical dystonia patients with GPi-DBS. 11 This pattern of functional changes was also shown to be intimately related to clinical improvement. This work highlights the potential for imaging biomarkers in DBS programming.

There is growing scientific evidence demonstrating the safety and efficacy of DBS for the treatment of medically refractory dystonia, however, optimal patient and surgical target selection remains unclear. Here multiple examples are presented that demonstrate how advances in neuroimaging are contributing to the understanding of DBS therapy in dystonia. These advances may lead to improved patient and target selection and DBS programming.
